# 378. De-escalating Vancomycin in the ICU: Results of the Swab Testing to Optimize Pneumonia treatment with empiric Vancomycin (STOP-Vanc) Randomized Controlled Trial

**DOI:** 10.1093/ofid/ofaf695.125

**Published:** 2026-01-11

**Authors:** Jeffrey Freiberg, Edward Qian, Kylie Nairon, Ben Ereshefsky, Cassandra Hennessy, Grace Van Winkle, Ariel Lewis, Joy Justice, Justin Siemann, Joanna Stollings, Taylor Rali, Frank Harrell, Cheryl Gatto, George E Nelson, Todd Rice

**Affiliations:** Vanderbilt University Medical Center, Nashville, TN; Vanderbilt University Medical Center, Nashville, TN; Vanderbilt University Medical Center, Nashville, TN; Vanderbilt University Medical Center, Nashville, TN; Vanderbilt University Medical Center, Nashville, TN; Vanderbilt University Medical Center, Nashville, TN; Vanderbilt University Medical Center, Nashville, TN; Vanderbilt University Medical Center, Nashville, TN; Vanderbilt University Medical Center, Nashville, TN; Vanderbilt University Medical Center, Nashville, TN; Vanderbilt University Medical Center, Nashville, TN; Vanderbilt University Medical Center, Nashville, TN; Vanderbilt University Medical Center, Nashville, TN; Vanderbilt University Medical Center, Nashville, TN; Vanderbilt University Medical Center, Nashville, TN

## Abstract

**Background:**

Methicillin-resistant *Staphylococcus aureus* (MRSA) is an infrequent, yet commonly feared cause of community-acquired pneumonia (CAP), leading to frequent use of empiric vancomycin. Multiple retrospective studies suggest the use of polymerase chain reaction (PCR) testing to detect MRSA on nasal swabs can be beneficial in guiding de-escalation of vancomycin in CAP treatment. However, there is a lack of randomized controlled trial (RCT) data evaluating the use of MRSA PCR testing for this purpose, and data regarding this use in an intensive care unit (ICU) are limited.

**Table 1.**
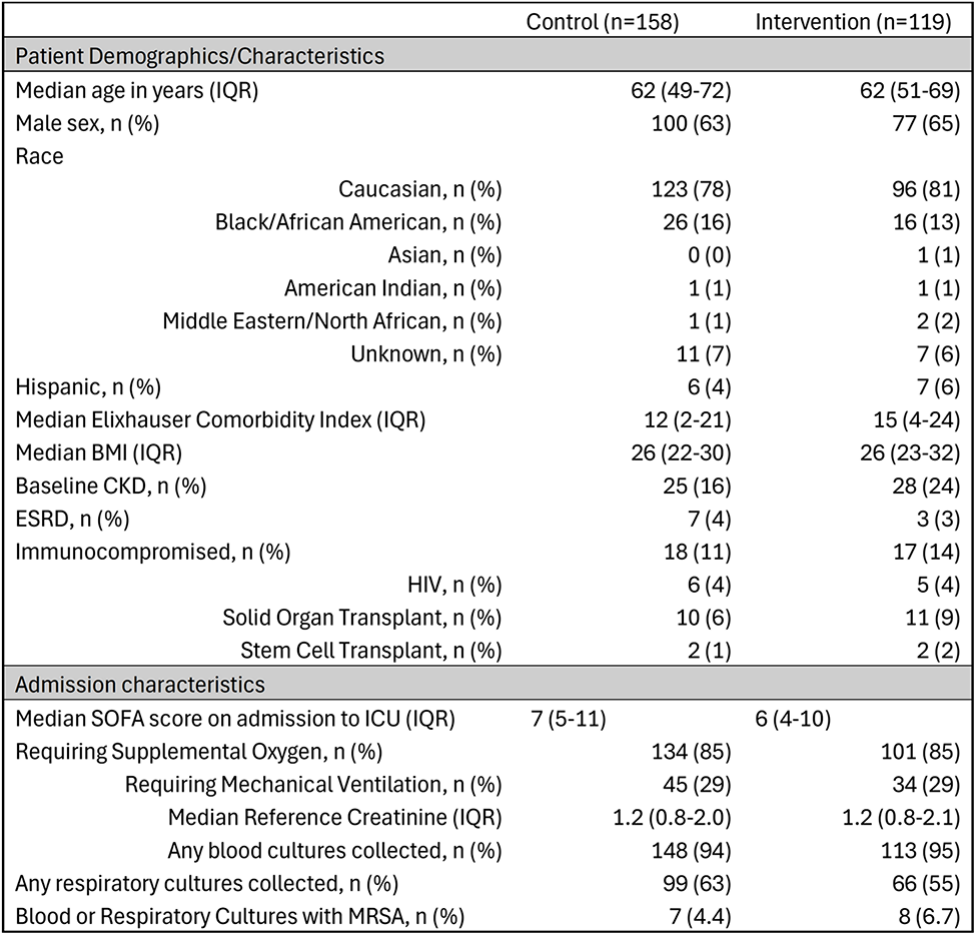
Baseline Patient Demographics and Characteristics

**Figure 1.**
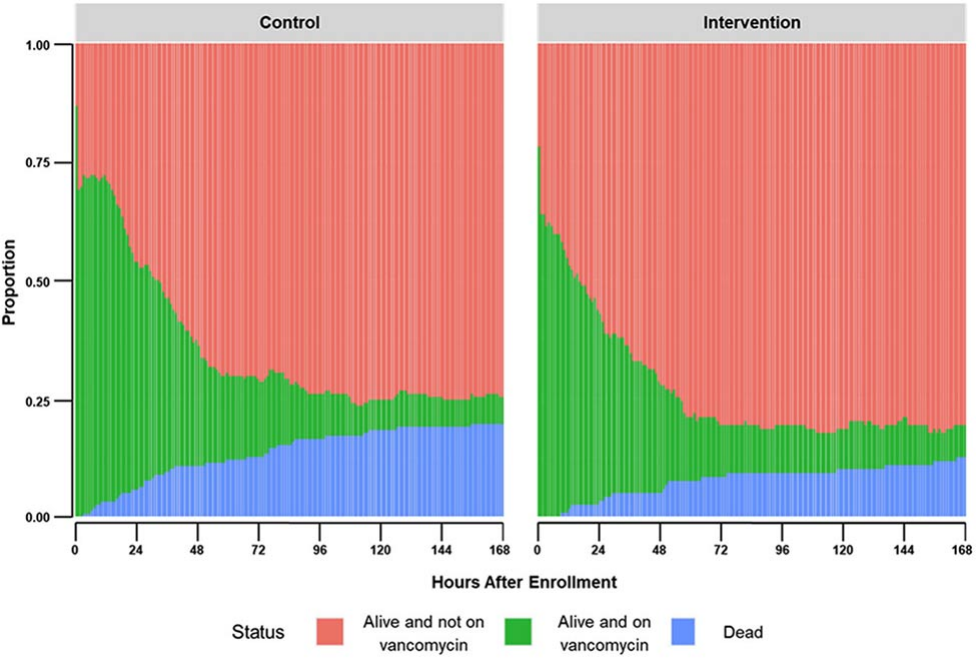
Proportion of Subjects Occupying a Given State Over Time Based on Treatment Arm

**Methods:**

STOP-Vanc is a pragmatic, prospective, single center, non-blinded randomized trial testing the effect of MRSA PCR testing on inappropriate vancomycin use in the ICU. Adult ICU patients with suspicion of CAP were randomized 1:1 to receive usual care either with (intervention) or without (control) the addition of MRSA nares PCR testing following ICU admission. The primary outcome was vancomycin-free hours alive, defined as the expected number of hours alive and free of vancomycin use within the first 7 days of trial enrollment as estimated using a longitudinal proportional odds ratio model adjusted for baseline covariates.

**Results:**

277 adult ICU patients were randomized between April 2024 and January 2025. Baseline patient demographics were similar between arms (Table 1) with an overall MRSA infection incidence of 5.4%. MRSA colonization was 17.8% in the intervention arm with MRSA nares PCR testing demonstrating a negative predictive value (NPV) of 0.989. Mean vancomycin-free hours alive was 109.4 in the intervention arm vs 105.1 in the control arm (difference 4.2, 95% CI -10.2 to 17.9). There was little evidence for differences in 30-day mortality (26% vs 35%; OR=0.70, 95% CI 0.41 to 1.20) or acute kidney injury (29% vs 32%; OR =0.93, 95% CI 0.54 to 1.60).

**Conclusion:**

Among patients with suspicion for CAP in an ICU with moderate rates of MRSA, the NPV of MRSA nasal swab PCR remains high. However, PCR testing showed little evidence of reducing vancomycin use in this study. While the use of MRSA PCR testing on ICU admission in patients with CAP should be used to guide vancomycin de-escalation, additional education and antimicrobial stewardship interventions are likely required to reduce vancomycin use in ICU settings.

**Disclosures:**

All Authors: No reported disclosures

